# Outcomes following extended postoperative recovery unit admission in noncardiac surgery: A systematic review and meta-analysis

**DOI:** 10.1097/EJA.0000000000002145

**Published:** 2025-03-06

**Authors:** Bernard R. Schockaert, René M. van Bruchem, Maarten F. Engel, Robert Jan Stolker, Felix van Lier, Sanne E. Hoeks

**Affiliations:** From the Clinical Research Group, Department of Anaesthesiology, Erasmus Medical Centre, Rotterdam, The Netherlands (BRS, RMvB, RJS, FvL, SEH), Department of Anaesthesiology and Intensive Care, AZ Delta, Roeselare, Belgium (BRS) and Erasmus Medical Centre, Rotterdam, The Netherlands (MFE)

## Abstract

**BACKGROUND:**

Surgery carries inherent risks, with the postoperative phase being as critical as the intraoperative period. Enhanced perioperative care units, positioned between general wards and intensive care units, aim to provide adequate postoperative management and resource allocation. Despite their widespread implementation, evidence on outcomes remains limited.

**OBJECTIVES:**

The primary outcome was 30-day or in-hospital mortality following extended postoperative recovery, with subgroup meta-analysis examining enhanced perioperative care units and intensive care units. Secondary outcomes included, among others, hospital length of stay.

**DESIGN:**

Systematic review with meta-analysis.

**DATA SOURCES:**

A comprehensive search was conducted in MEDLINE, Embase, Web of Science, Cochrane Central, and Google Scholar from inception up to 22 April 2024.

**ELIGIBILITY CRITERIA:**

The search string encompassed extended postoperative recovery units, including enhanced perioperative care units and intensive care units, for noncardiac, nontransplant surgery, excluding speciality-specific, age-specific, indirect and nonsurgical admissions. Two reviewers independently conducted screening, eligibility assessment and quality appraisal.

**RESULTS:**

Of 28 179 records screened, 24 were included of which 22 were unique studies. The overall pooled random-effects mortality, based on 15 studies, was 3 (95% confidence interval (CI) 2 to 6)%. Subgroup analysis demonstrated a mortality of 2 (95% CI 1 to 4)% for patients managed in enhanced perioperative care units and 8 (95% CI 4 to 14)% in intensive care units (*χ*^2^ = 7.99; *P* < 0.01). Risk of bias (ROBINS I) was moderate to serious, and heterogeneity substantial. Pooled hospital length of stay, based on six studies, was 8.6 (95% CI 5.9 to 11.3) days.

**CONCLUSION:**

Pooled mortality following extended postoperative recovery in noncardiac surgery was 3% (95% CI 2 to 6). Subgroup analysis indicated lower mortality among patients managed in enhanced perioperative care units. However, considerable heterogeneity in operational definitions, unit capabilities, and admission criteria necessitates cautious interpretation while reflecting real-world practices. Delineation through further research is warranted.

**PROSPERO REGISTRATION:**

CRD42023457051.

## Graphical abstract

**Figure:**
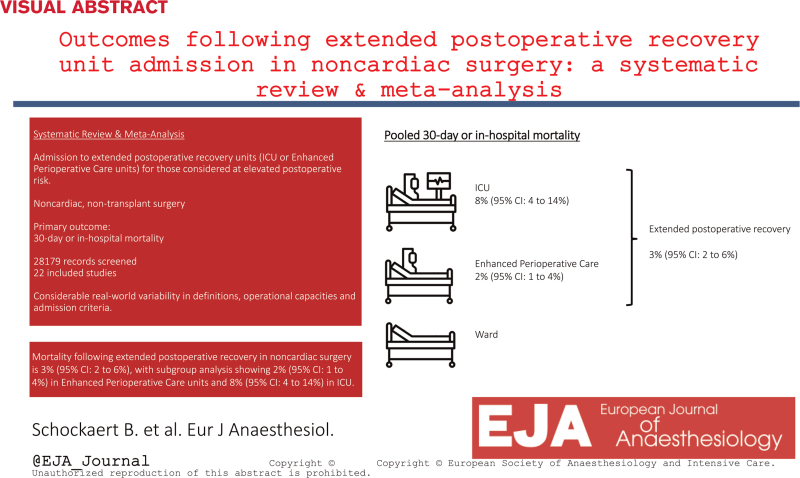

**KEY POINTS**

- A small subset of high-risk surgical patients accounts for most adverse outcomes.
- Enhanced perioperative care units bridge the gap between ward care and intensive care.
- Variability in operational capabilities and admission criteria reflects heterogeneity in real-world practice.
- Enhanced perioperative care units optimise resources while providing quality care.

## Introduction

Globally, millions of surgical procedures are conducted annually, with an estimated total of 312.9 million in 2012.^1^ Despite its intended clinical benefits, surgery carries inherent risks. Each year, approximately 4.2 million people die within 30 days postoperatively, accounting for 7.7% of global deaths and ranking it as the third leading cause of death after ischaemic heart disease and stroke.^2^ Mortality rates following major surgery range from 0.5 to 5%.^3^ Data from a European noncardiac surgery cohort reveal a postoperative in-hospital mortality rate of 4%, and an 8% critical care unit admission rate, with significant variations across countries. Notably, 73% of the fatalities had not been admitted to a critical care unit (CCU).^4^ Complications are a significant concern, affecting up to 25% of patients undergoing major surgery.^3^ Recent evidence supports these findings, showing that adverse events occur in over one-third of surgical cases, with almost half being classified as major and largely preventable.^5^

In accordance with the Pareto principle – the observation by Italian economist Vilfredo Pareto that a limited proportion (roughly 20%) is responsible for the majority of effects (approximately 80%) – patients in the highest risk quintile (20%) are responsible for around 90% of postsurgical deaths.^6^ This underscores the importance of optimal postoperative care, as undertriage is associated with increased mortality and morbidity.^7^ This phenomenon, often referred to as failure to rescue, highlights the need for timely recognition and effective management of complications.^8,9^

Patients at risk include those with preexisting medical conditions, undergoing complex surgery or encountering unexpected peri-operative and postoperative events. Optimal preoperative assessment, along with appropriate surgical and organisational strategies, are crucial in mitigating these risks.^10–13^ For such patients, a time-limited, direct postoperative admission to extended postoperative recovery units has been proposed as a beneficial approach. The scarcity of intensive care resources alongside logistical and financial challenges has led to the creation of dedicated postoperative units, also referred to as Enhanced Perioperative Care (EPC) units. They are known by various names and forms and deliver time-limited supportive care. They occupy a distinct position within the postoperative care continuum, bridging the gap between the intensive care unit (ICU) and general ward care.^14^ Characterised by enhanced medical and nursing staffing, they offer advanced monitoring and interventions beyond the capabilities of a general ward, though not to the full extent of ICU-level care. This delineation aligns with the framework outlined by the Faculty of Intensive Care Medicine/Centre for Perioperative Care Guidance on Establishing and Delivering Enhanced Perioperative Care Services.^15^ Initially described for cardiac surgery patients in the UK in 1986, these EPC services have since been widely adopted.^16^ Although this concept appears sound, evidence remains sparse and conflicting.^17,18^ This systematic review aims to synthesise the diverse terminologies into a cohesive framework referred to as extended postoperative recovery units, encompassing ICUs and the comprehensive concept of EPC units. Additionally, this review evaluates the outcomes of patients undergoing noncardiac surgery admitted to these units.

## Methods

This systematic review was conducted in strict adherence to the Preferred Reporting Items for Systematic Reviews and Meta-Analyses (PRISMA) guidelines and the PRISMA-S extension to the PRISMA Statement for Reporting Literature Searches in Systematic Reviews and was preregistered on PROSPERO (CRD42023457051).^19,20^

### Eligibility

The search strategy (Appendix 1) encompassed studies investigating noncardiac, nontransplant surgery. Articles explicitly including cardiac surgery or transplantations were excluded owing to their distinct risk profiles. Defining extended postoperative recovery units posed a challenge due to the heterogeneity in definitions, reflecting real-world clinical practice. The search string aimed at being as comprehensive as possible, identifying units with a shared objective of delivering extended postoperative recovery through advanced monitoring, enhanced medical and nursing staffing, and expanded intervention capabilities. This included both Level 2 and Level 3 postoperative care pathways.^21^ Our inclusion criteria covered a period of more than 6 h in the recovery room or post anaesthesia care unit (PACU), admissions to level-one areas, 23-h care units, transitional and intermediate care units, high dependency units (HDU), advanced recovery room care units (ARRC), high acuity units, overnight intensive recovery units and a CCU or ICU stay. To focus on the short-term postoperative recovery care, we excluded studies with intended median ICU stays exceeding 48 h, guided by the 2020 UK guideline, suggesting targeted and time-limited interventions with discharge aimed within 24 h, with only a small proportion remaining for a maximum of 48 to 72 h.^15^ Additionally, highly specialised ICUs, such as neonatal (NICU) and paediatric (PICU) intensive care units, were excluded due to their exclusive focus on specific patient populations. On the other hand, apart from patients younger than 28 days, children defined as those less than 18 years were not excluded if part of a broad and general admitted population. Only direct postoperative admissions were included thereby excluding specialised postoperative step-up or step-down units that admitted patients from a lower or higher level of care. Nonsurgical admissions, referring to admissions for medical conditions or procedures that were not directly related to surgical interventions, were excluded. Noncardiac surgery studies had to involve at least three different specialities, thereby broadening the patient population studied and reflecting the comprehensive care provided in extended postoperative recovery units, as compared to speciality-specific units. All clinical outcomes were included due to their potential relevance.

### Literature search strategy

An exhaustive search strategy was developed by an information specialist (ME) in cooperation with the lead author (BS). The search was developed in Embase.com, optimised for sensitivity and then translated to other databases.^22^ The search was conducted from inception to 22 April 2024 across the databases MEDLINE ALL via Ovid, Embase.com, Web of Science Core Collection and the Cochrane Central Register of Controlled Trials via Wiley. A supplementary search in Google Scholar retrieved the 200 highest ranked references using Publish or Perish software.^23^ The original search in June 2023 was updated back from inception on 22 April 2024 to include additional relevant terms.^24^ Search strategies for MEDLINE and Embase incorporated relevant thesaurus terms from Medical Subject Headings (MeSH) and Emtree, with terms searched in titles, abstracts and keywords. Boolean and proximity operators combined terms for PACU and outcome. Searches excluded conference abstracts, case reports, animal-only, non-English and ambulatory surgery-focused articles. Reference lists of key review articles and included studies were scanned for additional relevant articles.^25^ Duplicates were removed in Endnote 20 Version, 2013, Clarivate, Philadelphia PA by an experienced information specialist (ME).^26^

### Study selection and data extraction

Study selection was conducted independently by two researchers (BS, RB). In cases of disagreement or ambiguity, consultation with experts (SH, FL) was sought to reach a consensus. This process was executed through a systematic, stepwise approach involving title review, abstract screening, full-text retrieval and eligibility assessment. Exclusions were detailed and presented in the PRISMA flow diagram. Only ethically compliant studies were included. We employed Endnote and Covidence systematic review software, Veritas Health Innovation, Melbourne, Australia (available at www.covidence.org) for structured screening, retrieval and eligibility assessment. Data extracted from the included articles, using the aforementioned software, encompassed the author, publication year, location, study design, name of the extended postoperative recovery unit, study population, admission criteria and measured outcomes.

### Risk of bias

The risk of bias was assessed using the Cochrane Risk of Bias 2 tool for Randomised Controlled Trials (RCT).^27^ The Risk Of Bias In Non-Randomised Studies – of Interventions (ROBINS-I) tool was used for nonrandomised studies.^28^ If direct comparison is feasible, the Grading of Recommendations, Assessment, Development, and Evaluations (GRADE) method will be applied.^29^

### Primary outcome

The primary outcome was short-term mortality, defined as 30-day or in-hospital mortality, based on the data available in the included studies. Additional data were requested from the authors when necessary. Subgroup analysis was conducted to address expected heterogeneity rather than comparing resource allocation. Where pooling was not feasible, mortality outcomes were presented narratively.

### Additional outcomes

Hospital length of stay (hLOS) was presented separately and, if feasible, pooled. Other outcomes were presented descriptively.

### Statistical and data analysis

A meta-analysis was conducted if more than one study was available. Random-effects models were used, as considerable heterogeneity was expected. Between-study variability was assessed using the *I*^2^ statistic, with *τ*^2^ serving as an additional estimator to address heterogeneity. The primary outcome, mortality, was pooled using a generalised linear mixed model with logit-transformed proportions. This approach was chosen over the inverse variance method because of its capability in addressing between-study heterogeneity. All studies reporting mortality were included in the overall calculation for pooled proportions. Furthermore, subgroup analysis was performed for patients receiving care in EPC units and in ICUs. Results of the meta-analysis were presented as pooled proportions with corresponding 95% confidence intervals and a prediction interval. A 95% prediction interval was included to estimate the range of true effects in future settings. Forest plots were generated to display pooled estimates, individual study effects and subgroup analyses.

To estimate the pooled duration of hLOS, a meta-analysis was conducted using the median-based method ‘quantile estimation’ within the ‘metamedian’ package, suitable for heterogeneous data.^30^ Study-specific estimates were combined in a pooled median, with 95% confidence intervals.

A post hoc pooled comparative meta-analysis was performed using data of studies that directly compared 30-day mortality between patients managed in EPC units with those in ward settings. The analysis was performed using the metabin function in R, which synthesises binary outcomes from multiple studies. The pooling of data was conducted using the inverse variance method, with odds ratios (ORs) as the summary measure. A random-effects model was employed to account for both within-study variability and between-study heterogeneity. Only studies that included these direct comparisons were included in the analysis.

The significance level was set at *P* less than 0.05. All statistical analyses were done using RStudio Team [RStudio 2024.040: Integrated Development for R. RStudio, PBC, Boston, Massachusetts, USA (packages ‘meta’, “metaprop’, ‘metafor’, ‘metamedian’ and ‘metareg’)].

## Results

### Study selection and characteristics

**Fig. 1:**
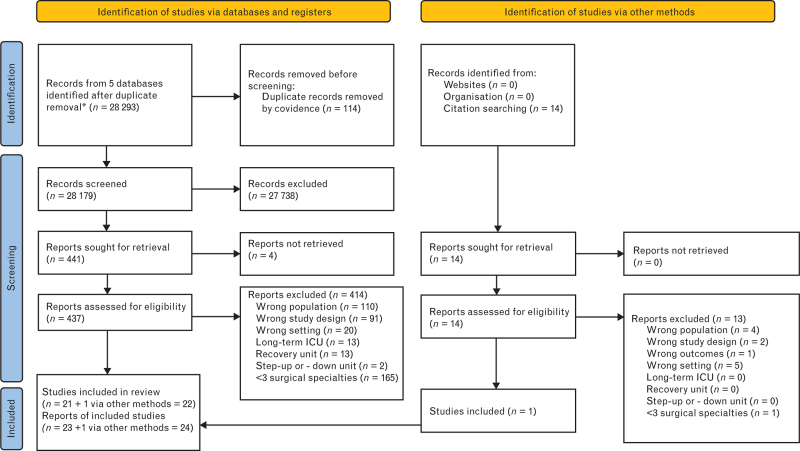
PRISMA 2020 flow diagram for new systematic reviews which included searches of databases, registers and other sources.^19,20^

^∗^ Due to an update during the process, more records were screened than the sum of the following^24^: MEDLINE (*n* = 17 557), Embase (*n* = 7594), Web of Science Core Collection (*n* = 1598), the Cochrane Central Register of Controlled Trials (*n* = 909), the top 200 ranked articles on Google Scholar (*n* = 63)

A total of 28 179 unique records were identified from five databases. Of these, 21 studies were included, and one study was identified through citation searching.^4,17,31–50^ Of note, two additional reports were derived from conducted studies as illustrated in the PRISMA flow chart (Fig. 1).^51,52^

Tables 1 and 2 summarise the 22 included studies, which exhibited notable heterogeneity. None of the studies were RCTs. Eight (36%) were multicentre and 13 (59%) were retrospective studies.

**Table 1:** Characteristics of the 22 included studies

Authors	Year of publication	Location	Study design (as described in article)	Name of the extended postoperative recovery unit
Costa-pinto *et al*.^31^	2022	Australia	Single centre retrospective observational	RHD
Davies *et al*.^32^	1999	United Kingdom	Single centre retrospective before-and-after	HDU
Harmse *et al*.^33^	2023	South Africa	Single centre retrospective observational	PAHCU
Heller *et al*.^34^	2008	United Kingdom	Single centre retrospective	POSU
Irone *et al*.^35^	2002	Italy	Single centre prospective observational	PACU
Jerath *et al*.^36^	2018	Canada	Multicentre (93 hospitals) prospective observational cohort	ICU
Jerath *et al*.^37^	2020	Canada	Multicentre retrospective cohort	ICU
Jhanji *et al*.^38^	2008	United Kingdom	Multicentre prospective observational	CCU
Koning *et al*.^39^	2024	Netherlands	Single centre retrospective observational interrupted time series	PACU
Ludbrook *et al*.^41^	2023	Australia	Single centre prospective observational cohort	ARRC
Ludbrook *et al*.^40^	2021	Australia	Multicentre prospective feasibility before-and-after	ARRC
McIlroy *et al*.^42^	2006	Australia	Single centre prospective observational	HDU
Paw *et al*.^50^	2000	United Kingdom	Single centre prospective observational	HDU
Pearse *et al*.^4^	2012	Europe	Multicentre prospective cohort	CC
Prin *et al*.^43^	2015	United Kingdom	Multicentre retrospective observational cohort	HDU
Rosenthal *et al*.^44^	1998	USA	Multicentre retrospective cohort	ICU
Thevathasan *et al*.^45^	2019	USA	Single centre observational propensity matched cohort	ICU
Turner *et al*.^46^	1999	United Kingdom	Single centre prospective observational	HDU
Uzman *et al*.^47^	2016	Turkey	Single centre retrospective observational	ICU
van Tunen *et al*.^48^	2020	Netherlands	Single centre retrospective cohort	PACU
Wickboldt *et al*.^49^	2015	Switzerland	Single centre retrospective observational	IMC
Wunsch *et al*.^17^	2016	USA	Multicentre retrospective observational	ICU

ARRC, advanced recovery room care; CC, critical care; CCU, critical care unit; HDU, high dependency unit; ICU, intensive care unit; IMC, intermediate care unit; PACU, post-anaesthesia care unit; PAHCU, post-anaesthesia high-care unit; POSU, postoperative surgical unit; RHD, recovery high dependency unit; USA, United States of America.

**Table 2:** All 24 reports based on 22 studies describing the study population (*n*), admission criteria and respectively measured outcomes

Authors	Study population (n)	Admission criteria	Outcomes (primary outcome) respectively
Costa-pinto *et al*.^31^	1171 (86 of the 1257 were not primarily surgical RHD admissions)	Based on clinical judgement	Median RHD stay 12.6 [9.1 to 15.9] h; hLOS 8.3 [5.0 to 17.3] days; non-invasive ventilation 4.7%; vasopressor support 23.1%; ICU admission 13%; Medical Emergency Team call within 24 hours of discharge from RHD 6.4%; out of 37 RHD patients who died, 5 were not primarily surgical RHD admissions and were therefore excluded
Davies *et al*.^32^	300 (overall surgical hospital admissions pre-HDU 4471 vs 5154 HDU)	Patients requiring invasive monitoring with a predicted LOS less than 48h and single organ failure (excluding catecholamine support or (non-) invasive ventilation)	Pre-HDU vs post-HDU: overall in-patient hospital mortality 2.16% vs 3.2%; ICU admission 1.7% vs 1.3%; ICU LOS 4.5 vs 5.6 days; ICU surgical patient mortality 29% vs 58%; HDU 30-d mortality 13% There was a 15% increase in surgical workload (defined as total number of surgical admissions) between the two periods: Pre-HDU 4471 vs post-HDU 5154. This resulted in a 12% increase in out-of-hours surgery
Harmse *et al*.^33^	1020	Based on clinical judgement	Use of the unit 87.2% pre-planned admissions; 6.76% exceeded 24h PAHCU stay; 1.07% ICU admissions; OR >1 to exceed 24-hour PAHCU stay: aortic stenosis, age <40y and age >61y, epidural care, and haemodynamic monitoring; 1 patient died in-hospital
Heller *et al*.^34^	843	Based on clinical judgement by anaesthetist and surgeon after major elective surgery, excluding invasive ventilation	Surgery cancellation ratio before POSU 186 of 503 and after POSU 34 of 843 patients; POSU mortality 2%
Irone *et al*.^35^	1142	Not described	Nine Equivalent of nursing Manpower Score (NEMS) 73.1% in high treatment level patients; hospital mortality 5.6%; mean PACU LOS 1 ± 0.8 days; mean Work Utilisation Ratio (WUR) 1.12 ± 0.21
Jerath *et al*.^36^	52 063 >40y old patients	Major elective noncardiac surgery (13 types)	Early admission to ICU (within 24h) in 52 063 (9.6%) of 541 524 major noncardiac procedures; 30-day MR of 2.4%; 90-day MR of 4.2%; median postoperative hLOS of 6 [4 to 9] days
Jerath *et al*.^37^	91 950^a^ >40 y old patients	Not described; 3 types of surgery studied: nephrectomy, gastrointestinal and peripheral arterial surgery	Higher hospital-specific ICU use (represented per quartile) was not associated with increased DAH30-90-180 or 30-day mortality
Jhanji *et al*.^38^	1470	High risk (predicted mortality >5%) and low risk unplanned ICU admission excluding cardiothoracic, neurosurgery, endoscopy, day case, transplantation, obstetric and burn management	Incidence of high-risk noncardiac procedures is 9.3% with overall MR of 12.2%, median hLOS 16 [9 to 30] days; 35.3% of high-risk patients were admitted to CCU; 294 died of which only 49% were admitted to a CCU
Koning *et al*.^39^	625	VATS, (para)thyroidectomy, open lobectomy, EVAR, endoscopic low GIS surgery, cystectomy, debulking ovarian, hip fracture, revision knee or hip, arterial revascularization or embolectomy lower limb	IMCU vs PACU period: median hLOS IMCU 7.2 [4.2 to 12.0] days vs PACU 6.0 [3.6 to 9.1] days, *P* < 0.001; geometric mean ratio of PACU hLOS 0.77 (0.66 to 0.91), *P* = 0.002; no difference in postoperative complications or ICU admissions
Ludbrook *et al*.^41^ Leaman *et al*.^51^ (same study)	447 ARRC vs 407 usual care	Noncardiac surgery, anticipated hospital stay >1 night with medium risk (30-day mortality 0,7-5% based on NSQIP)	DAH 30D ARRC vs usual care: mean 17 ± 11 days vs 15 ± 11 days, *P* = 0.04; <24h MER-level complications in ARRC 43 (12.4%) vs 13 (3.7%), *P* < 0.001; >24h 9 (2.6%) vs 22 (6.3%), *P* = 0.03; no significant difference in hLOS, hospital readmissions, emergency department visits nor mortality ARRC +4.3 DAH 90d and decrease of 1081$ overall hospital cost per patient
Ludbrook *et al*.^40^ Lloyd *et al*.^52^ (same study)	95 before, 71 after	Moderate-risk patients (NSQIP predicted 30-day mortality of 1–4%) undergoing noncardiac surgery and who were scheduled for postoperative ward care	Feasible recruitment and postoperative follow-up of a total of 120 patients; 31 met MERT criteria; incidence of unplanned ICU admissions from ward 7 vs 1; quality of recovery scores was lower before ARRC; 90-day mortality RAH 3 vs 6; readmission within 90 days in RAH & PMCC 31 vs 17 24-48h after surgery MET-level events 4.6% before and 1.9% after
McIlroy *et al*.^42^	50 HDU vs 37 ward	Based on clinical judgement	Longer hLOS in HDU group: log hLOS HDU 14.3 ± 2.2 vs non-HDU 8.1 ± 3.2 days, *P* = 0.007; 30-d mortality 1 vs 1; no increase in adverse events
Paw *et al*.^50^	116 HDU vs 384 ward	Based on clinical judgement by anaesthetist; pre-operative assessment or unexpected perioperative events	HDU patients have higher POSSUM scores and higher complication rates within 48 hours after major surgery than ward care patients. Two patients died in each group.
Pearse *et al*.^4^	3580 critical care admissions >16y old	Inpatient (non-)elective surgery excluding day case, cardiac, neuro, radiological and obstetric procedures; critical care admission criteria not described	4% in-hospital mortality; 8% admission to critical care after surgery; 3580 critical care admissions of which 503 died in-hospital; CCU LOS 1.2 [0.9 to 3.6] days
Prin *et al*.^43^	3716 Postoperative HDU subgroup	Not described	LOS HDU 1.3 [0.9 to 2.8] days; total hospital LOS 11 [6 to 21] days; mortality in HDU 47 (1.3%); ICU admission from HDU 143 (3.9%)
Rosenthal *et al*.^44^	41 716 >16y old postoperative	Not described; postoperative ICU admissions excluding cardiac surgery, burn injuries, ICU admission for haemodialysis, ICU less than 4h, exitus within 4h of ICU admission	23.6% of postoperative ICU admissions were categorised as low severity (predicted mortality <1%); low severity ICU LOS 2 [2 to 3] days, low severity hLOS 6 [4 to 9] days vs higher 11 [8 to 18] days; in-hospital mortality low severity 33 (0.3%) vs higher 2482 (7.8%)
Thevathasan *et al*.^45^	3530	Individual preferences of anaesthesiologists and surgeons; propensity-matched cohort differed postop ICU or surgical ward in intermediate-risk surgery, 3 cohorts (first, second and third) respectively low medium and high propensity / need for ICU	Low propensity for ICU has an increased postoperative hLOS IRR 1.69 (1.59 to 1.79), *P* < 0.001 vs high propensity IRR 0.90 (0.85 to 0.95), *P* < 0.001; hospital costs low propensity IRR 1.92 (1.81 to 2.03), *P* < 0.001 vs high IRR 0.92 (0.88 to 0.97), *P* = 0.001
Turner *et al*.^46^	93	Based on clinical judgement by anaesthetist and surgeon. Major surgery (excluding minor day-case, gynaecological and elective orthopaedic surgery)	Optimal postoperative care unit (HDU or ITU) requested by anaesthetist and surgeon: 21 of the 132 ITU requests not met; 256 of the 349 HDU requests not met; overall mortality 1.5%; mortality for those with optimal postoperative care 1.2% vs suboptimal 3.1% (*P* < 0.038)
Uzman *et al*.^47^	1756	Based on clinical judgement	In-hospital mortality 256 (14.6%); median ICU stay 1 day (min 1 – max 418)
van Tunen *et al*.^48^	479	Based on clinical judgement	Off-target pre-operative PACU planning in 29%; secondary & tertiary PACU or ICU admissions in 31 patients (6%); 1.3% 30-d mortality (n = 6)
Wickboldt *et al*.^49^	4300	Based on clinical judgement (ASA PS, surgical risk, medical postoperative triage in recovery / PACU)	30-d in-hospital mortality 2.3% (n = 97); significant multivariate OR: age (years) 1.05 (1.03 to 1.08), *P* < 0.0001; emergency 3.42 (1.75 to 6.67), *P* < 0.001; abdominal surgery 2.91 (1.27 to 6.70), *P* = 0.012
Wunsch *et al*.^17^	129 227^b^ >65y old	Not described	There is no consistent relationship across 5 major surgical procedures (open or endovascular AAA, cystectomy, pancreaticoduodenectomy, oesophagectomy) between 3 ICU admission groups (low <50%, medium 50-89% and high ≥90%) and in-hospital mortality, hLOS or hospital costs

Data are mean ± SD, median [IQR], Odds Ratio (95% Confidence Intervals), and n (%) ARRC, Advanced Recovery Room Care); CCU, Critical Care Unit); DAH, Days Alive and out of hospital (or Days Alive and At Home)); EPC, Enhanced Perioperative Care, (I)); HDU, High Dependency Unit); ICU, Intensive Care Unit); IMCU, InterMediate Care Unit); ITU, Intensive Therapy Unit); LOS, (hospital) Length Of Stay); MER, Medical Emergency Response); MR, Mortality Rate); ns, non-significant); OR, Odds Ratio); PACU, Post-Anaesthesia Care Unit); PAHCU, Post-Anaesthesia High-Care Unit); PMCC, Peter Maccallum Cancer Centre); POSSUM, Physiological and Operative Severity Score for the enumeration of Mortality and Morbidity); POSU, PostOperative Surgical Unit); RAH, Royal Adelaide Hospital); RHD, Recovery High Dependency unit, (h); RR, (Incidence) Rate Ratio); SAEs, Severe Adverse Events). ^a^total cohort; ^b^total study population.

The nomenclature of the extended postoperative recovery units was diverse. Eight studies admitted patients to an intensive care or critical care unit.^4,17,36–38,44,45,47^ High dependency unit was used five times.^32,42,43,46,50^ Post anaesthesia care unit was used three times.^35,39,48^ Advanced recovery room care was used twice.^40,41^ Post anaesthesia high care unit, recovery high dependency unit, intermediate care unit and post operative surgical unit were used each once. ^31,33,34,49^

Study populations were nonuniform. Most records studied adult patients with four studies mentioning specific age populations: two above 40 years, one above 65 years and two on patients above 16 years.^4,17,36,37,44^ Admission criteria were stated in 12 studies. Nine studies admitted patients based on clinical judgement.^31,33,34,42,46–49,50^ One study used type of surgery as the primary admission criterion, and two used predicted mortality.^39,40,41^

### Risk of bias

**Fig. 2:**
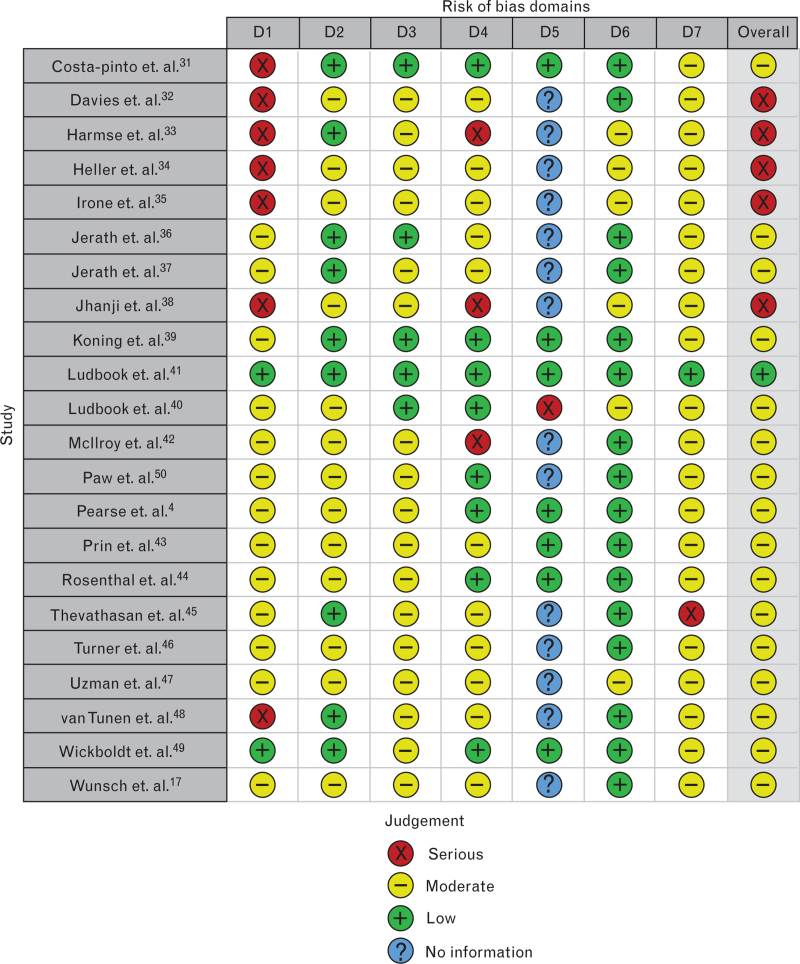
Risk of bias in non-randomised studies - of interventions (ROBINS-I).

Due to the absence of RCTs, ROBINS-I assessment was performed (Fig. 2), with additional details provided in Appendix 2. One study demonstrated an overall low risk of bias, while 16 showed a moderate risk, and five exhibited a high risk. Notable serious biases included confounding in seven studies especially due to preintervention factors.^31–35,38,48^ Deviations from intended interventions were identified in three studies due to institutional constraints.^33,38,42^ Missing data was identified in one study due to the exclusion of one of three participating centres, and selection of reported results was found in another study.^40,45^ Additionally, 14 studies lacked sufficient information concerning missing data.^17,32–38,42,45–48,50^

Domains: 1, bias due to confounding; 2, bias in the selection of participants; 3, bias in the classification of interventions; 4, bias due to deviations from intended interventions; 5, bias due to missing data; 6, bias in the measurement of outcomes; 7, bias in the selection of the reported result; judgement: serious, moderate, low, no information.

### Primary outcome

**Fig. 3:**
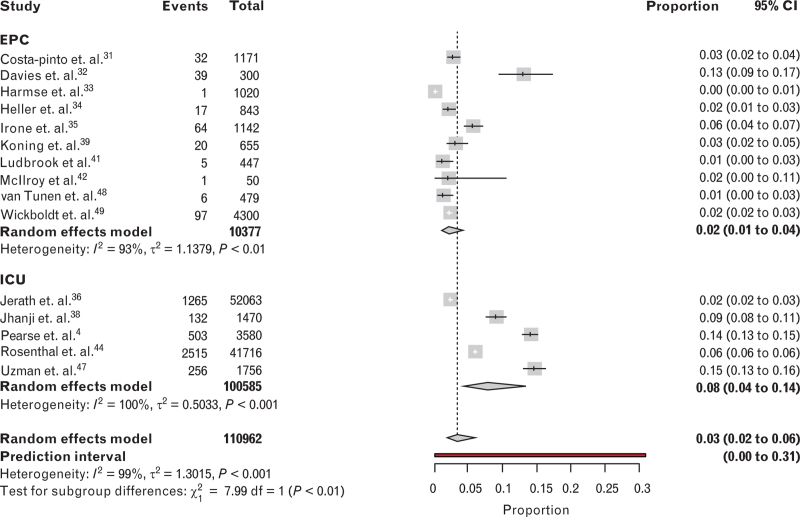
Mortality analysis (30-day or in-hospital mortality) with subgroup analysis: EPC and ICU.

Short-term mortality, defined as 30-day or in-hospital mortality, was reported across 15 studies, resulting in an overall pooled mortality of 3 (95% CI, 2 to 6)% with a prediction interval of 0 to 31%. Subgroup analysis demonstrated a mortality of 2 (95% CI, 1 to 4)% in patients managed in EPC units and 8 (95% CI, 4 to 14)% in ICUs. Subgroup difference testing resulted in *χ*^2^ = 7.99 with *P* less than 0.01 (Fig. 3). Statistical heterogeneity was high in both groups, with respectively *I*^2^ values of 93 and 100% and *τ*^2^ values of 1.14 and 0.50. Each subgroup included studies with relatively high mortality and relatively low mortality.^32,33,36,47^ Any additional data requests or clarifications made by the authors are depicted in Table 2.

Among the 22 studies included in the review, seven were excluded from the pooled mortality analysis due to the lack of applicable data. In one study, the authors reported the odds ratio (OR) for mortality divided into four quartiles, finding no adjusted association between hospital-specific ICU admission rate quartiles and Days Alive and at Home at 30-days (DAH30) or 30-day mortality risk.^37^ A before-and-after study indicated that it is feasible to conduct an RCT to investigate outcomes in patients admitted to an ARRC based on a predicted 30-day mortality of 1 to 4%.^40^ Another study on medical and surgical HDUs described an in-HDU mortality of 47 out of 3716 (1.3%) patients in a subgroup analysis of postoperative patients.^43^ Another record reported an overall mortality of 1.5% in major surgery patients receiving enhanced postoperative care (ICU and HDU). Patients who received the desired postoperative care had a mortality rate of 1.2%, compared to 3.1% in those who did not receive the requested level of care.^46^ Others found little consensus on ICU necessity after major surgery.^17^ They observed no consistent relationship between ICU usage and hospital mortality, hLOS, or cost of care across various surgical procedures and hospital ICU admission policies. Conversely, a propensity-matched cohort study suggested that postoperative intensive care might negatively impact postoperative hLOS and costs.^45^ However, patients with a high propensity for postoperative ICU admission showed an overall shorter hLOS and cost reduction.

### Additional outcomes

Of the 10 studies reporting hLOS, six were eligible for pooling as they provided either mean values with standard deviation or median values with interquartile ranges.^31,36,39,41–43^ Pooled hLOS was 8.6 (95% CI, 5.9 to 11.3) days (Appendix 3). Pooling was not feasible for four studies, as their data were subdivided into noncomparable groups. One study analysed three hospitals separately, and two of them (the Royal Adelaide and The Peter MacCallum Cancer Centre) reported a mean hLOS before and after ARRC implementation of 9.2 and 9.2, and 7.6 and 10.9 days, respectively.^40^ Whereas another study reported a median hLOS of 6 days for low-severity patients (APACHE III expected in-hospital mortality <1%) and 11 days for those with higher severity.^44^ Two studies subdivided hLOS into elective and nonelective surgery categories, observing longer stays in the nonelective group.^4,38^ All other measured outcomes are described in Table 2.

**Fig. 4:**
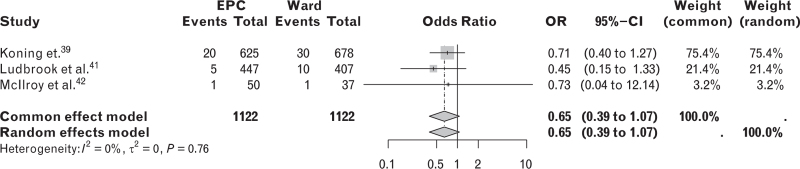
Post-hoc pooled analysis comparing mortality between patients managed in EPC units vs ward.

A post hoc pooled comparative analysis of studies investigating 30-day mortality between patients managed in EPC units and those in ward settings was conducted using data from three studies: one interrupted time series, one propensity score analysis, and one study comparing ward care patients who ideally would have been admitted to a high dependency unit.^39,41,42^ A random effects model showed a mortality OR for the EPC group of 0.65 (95% CI, 0.39 to 1.07) (Fig. 4).

## Discussion

This systematic review reports an overall pooled mortality of 3 (95% CI, 2 to 6)% following extended postoperative recovery after noncardiac, nontransplant surgery. Patients managed in EPC units exhibited a mortality of 2 (95% CI, 1 to 4)% and those admitted to the ICU exhibited a mortality of 8 (95% CI, 4 to 14)%. The mean hospital length of stay following admission to either EPC or ICU was 8.6 (95% CI, 5.9 to 11.3).

Admission to the EPC unit or ICU often relies on subjective clinical judgement, considering factors, such as preoperative health conditions, surgical risk or (un)expected perioperative needs.^53^ To standardise admission criteria, the UK guidelines published in 2020 recommend that noncardiac surgery patients with a predicted 30-day mortality of more than 1% but less than 5% may benefit from EPC services.^15^ Which corresponds to the 95% confidence interval of 1 to 4% mortality for EPC unit care reported in this review. Notably, two studies admitted patients to EPC units based on predicted mortality.^40,41^ Although mortality is a multifactorial outcome, the utilisation of validated prediction models, such as the National Surgical Quality Improvement Program (NSQIP), Surgical APGAR Score, Surgical Mortality Probability Model (SMPM), and Surgical Outcome Risk Tool (SORT), may aid decision-making, similar to the EuroSCORE II in cardiac surgery.^54–57^ It is noteworthy that cardiac surgery, given its high-risk nature and standard enhanced postoperative care, exhibits an observed 30-day mortality of 2%.^58,59^

The impact of extended postoperative recovery on mortality remains uncertain.^60^ To date, only one RCT, The Incare trial, has assessed the potential role of EPC unit admission for patients undergoing emergency abdominal surgery, a high-risk group.^18^ However, the trial was prematurely terminated due to slow recruitment and lower than expected mortality, yielding nonsignificant results. Consequently, the evidence relies mostly on observational data. Although outcomes appear to depend more on the early recognition and management of complications than on complication rates themselves, increasing admissions to extended postoperative recovery units has not been associated with improved outcomes.^9,17,36^ This trend holds true even in distinct high-risk surgical populations, as demonstrated by the STARSurg Collaborative group in major gastrointestinal and liver surgeries.^14^ Similarly, considerable variation in ICU admission criteria across countries and surgical types, including cardiac surgery, has been documented, with no survival benefit demonstrated.^61^ The lack of observed benefit may partly stem from three factors. First, for the vast majority of postoperative patients, care requirements overlap between the ward and extended postoperative recovery units. Second, complications often follow a time-dependent trajectory, with some arising after an initially uneventful recovery once patients have transitioned to the ward. Finally, admitting intermediate-risk patients to EPC units instead of the ICU may negatively affect outcomes in both groups, consistent with the Will Rogers effect or stage migration.

In contrast to the current systematic review and meta-analysis, two previous reviews narratively described enhanced postoperative recovery care, covering studies up until 2017 and 2018.^62,63^ One of these reviews explored the comparison between two-level and three-level care pathways, while both encompassed a broader range of patient populations, including those undergoing cardiac surgery, transplant procedures, and care in step-up/step-down units, as well as speciality-specific care. In contrast, our review incorporated more recent evidence and applied stricter inclusion criteria.

Several limitations must be considered when interpreting these findings. Substantial heterogeneity was observed among the included studies, arising from methodological variations, such as prospective versus retrospective designs and differing outcome measures, as well as statistical differences. Clinical discrepancies, particularly in the definitions of extended postoperative recovery units and their admission criteria, further added to this variability. Random-effects models and subgroup analyses were employed to partially address these challenges. However, the inherent differences in methodologies, statistical approaches and real-world practices could not be fully mitigated, leading to greater uncertainty in the results. These limitations, along with the use of the Generalised Linear Mixed Model for the proportions, may have contributed to the wide prediction interval. Furthermore, temporal evolutions in perioperative practices may have influenced the measured outcomes. Although all age groups were included (except neonates) to mitigate age-related bias, no studies specifically addressing paediatric EPC units were identified. The risk of bias in most included studies was moderate to serious, as assessed by the ROBINS-I tool, potentially impacting the robustness. Only studies involving three or more surgical specialities were included, resulting in the exclusion of 165 reports (Fig. 1). This criterion was chosen to emphasise the broad applicability of extended postoperative recovery care, which is typically managed by anaesthesiologists or intensivists and addresses a wider range of postoperative patients’ needs beyond speciality-specific concerns and speciality-specific patients’ comorbidities. This approach is consistent with clinical practice in ICUs, where noncardiac surgical care is rarely subclassified. While this selection criterion aimed to reduce bias from speciality-specific care, it may have nonetheless influenced the results. A median length of stay exceeding 48 h in an extended postoperative recovery unit was chosen as cut-off value in accordance with the 2020 UK guideline. While this threshold was intended to minimise bias from outliers exceeding the scope of limited and high-turnover care units, it may inadvertently introduce bias. The pooled hLOS must be interpreted with caution because of differences in reporting metrics and the limited number of studies. Lastly, the analysis was limited by an insufficient number of studies to allow differentiation between subcategories, including institutional variations, technical capabilities, elective versus nonelective surgeries and specific comorbidities.

## Conclusion

This review found an overall pooled mortality of 3% (95% CI, 2 to 6) after extended postoperative recovery in noncardiac surgery, with lower mortality observed among patients managed in EPC units. However, considerable variability in the definitions, operational capacities and admission criteria warrants careful interpretation. This emphasises the need for standardisation and future research, while also acknowledging the inherent diversity of clinical practices across different healthcare settings.

## Supplementary Material

Supplemental Digital Content

## Supplementary Material

Supplemental Digital Content

## Supplementary Material

Supplemental Digital Content
