# Supplementary material for: Outcomes following extended postoperative recovery unit admission in noncardiac surgery: A systematic review and meta-analysis
Source: Eur J Anaesthesiol. 2025 Mar 6;42(5):407–18. doi: 10.1097/EJA.0000000000002145 (PMC11970609; doi:10.1097/EJA.0000000000002145)
Supplement: Supplemental Digital Content [file ejanet-42-407-s001.docx]

Appendix 1: 20240422

Post anesthesia care unit

SR

*Update 22-4-2024: adding: IC use/ultilization en high acuity unit*

| **Database searched** | **Platform** | **Years of coverage** | **Records** | **Records after duplicates removed** |
| --- | --- | --- | --- | --- |
| Medline ALL | Ovid | 1946 - Present | 17649 | 17557 |
| Embase | Embase.com | 1971 - Present | 22656 | 7594 |
| Web of Science Core Collection* | Web of Knowledge | 1975 - Present | 14241 | 1598 |
| Cochrane Central Register of Controlled Trials** | Wiley | 1992 - Present | 4345 | 909 |
| Additional Search Engines: Google Scholar*** (200 top-ranked) | | | 200 | 63 |
| **Total** | | | **59000** | **27721** |

*Science Citation Index Expanded (1975-present) ; Social Sciences Citation Index (1975-present) ; Arts & Humanities Citation Index (1975-present) ; Conference Proceedings Citation Index- Science (1990-present) ; Conference Proceedings Citation Index- Social Science & Humanities (1990-present) ; Emerging Sources Citation Index (2005-present)

** deleted abstracts from trial registries

***Google Scholar was searched via "Publish or Perish" to download the results in EndNote.

No other database limits were used than those specified in the search strategies

***new references:***

exclude trial registries from Cochrane

exclude conference abstracts and case reports

only human studies

only english

**Embase 22565**

('recovery room'/de OR 'surgical intensive care unit'/de OR 'high dependency unit'/de OR 'intermediate care unit'/de OR 'transitional care'/de OR (('intensive care'/mj/de OR 'intensive care unit'/mj/de OR 'surgical intensive care unit'/mj/de OR 'medical intensive care unit'/mj/de) AND ('health care utilization'/mj/de OR 'utilization review'/mj/de)) OR (((recovery) NEAR/3 (room*)) OR PACU OR ((postoperativ* OR post-operativ* OR postanaesth* OR postanesth* OR post-anaesth* OR post-anesth*) NEAR/6 (care OR recovery) NEAR/6 (unit* OR room* OR ward* OR beds OR setting* OR area* OR admission*)) OR ((extended OR prolong*) NEAR/6 (postoperativ* OR post-operativ* OR postanaesth* OR postanesth* OR post-anaesth* OR post-anesth*) NEAR/6 (recovery OR care OR beds OR setting* OR area* OR admission*)) OR ((high-dependenc* OR high-care*) NEXT/3 (unit* OR room* OR ward* OR beds OR setting* OR area* OR admission*)) OR ((surgical) NEXT/3 (intensive-care* OR IC) NEXT/3 (unit* OR ward* OR room* OR beds OR setting* OR area* OR admission*)) OR ((intermediate) NEAR/6 (care) NEAR/6 (unit* OR room* OR ward* OR beds OR setting* OR area* OR admission*)) OR IMCU* OR ((overnight) NEAR/6 (intensiv*) NEAR/6 (recovery)) OR ((23h* OR 23-h*) NEXT/2 (recovery)) OR level-1-area* OR level-one-area* OR transitional-care OR ((high-acuity) NEAR/3 (unit* OR room* OR ward* OR beds OR setting* OR area* OR care))):ab,ti,kw OR ((IC OR ICU OR intensive-care*) NEAR/3 (use OR utiliz* OR utilis*)):ti) **AND** ('treatment outcome'/exp OR mortality/exp OR morbidity/exp OR 'major adverse cardiac event'/de OR complication/exp OR complication:lnk OR 'intensive care'/de OR 'outcome assessment'/de OR reoperation/de OR 'length of stay'/de OR 'hospital discharge'/de OR (outcome* OR mortalit* OR morbidit* OR major-adverse-cardiac-event* OR MACE OR complication* OR ((ICU OR IC OR intensive-care* OR critical-care*) NEAR/3 (stay OR admi*)) OR reoperat* OR re-operat* OR length-of-stay* OR discharge* OR return-to-theat*):ab,ti,kw) NOT ([Conference Abstract]/lim OR [Conference Review]/lim) NOT ('case report'/de OR (case-report):ti) AND [ENGLISH]/lim NOT ([animals]/lim NOT [humans]/lim) NOT ('ambulatory surgery'/mj/de OR (((ambulator* OR day OR outpatient*) NEAR/3 (surg* OR operat*))):ti)

**Medline 17649**

(Recovery Room/ OR Transitional Care/ OR ((*Critical Care/ OR *Intensive Care Units/) AND (*"Facilities and Services Utilization"/ OR *Utilization Review/)) OR (((recovery) ADJ3 (room*)) OR PACU OR ((postoperativ* OR post-operativ* OR postanaesth* OR postanesth* OR post-anaesth* OR post-anesth*) ADJ6 (care OR recovery) ADJ6 (unit* OR room* OR ward* OR beds OR setting* OR area* OR admission*)) OR ((extended OR prolong*) ADJ6 (postoperativ* OR post-operativ* OR postanaesth* OR postanesth* OR post-anaesth* OR post-anesth*) ADJ6 (recovery OR care OR beds OR setting* OR area* OR admission*)) OR ((high-dependenc* OR high-care*) ADJ3 (unit* OR room* OR ward* OR beds OR setting* OR area* OR admission*)) OR ((surgical) ADJ3 (intensive-care* OR IC) ADJ3 (unit* OR ward* OR room* OR beds OR setting* OR area* OR admission*)) OR ((intermediate) ADJ6 (care) ADJ6 (unit* OR room* OR ward* OR beds OR setting* OR area* OR admission*)) OR IMCU* OR ((overnight) ADJ6 (intensiv*) ADJ6 (recovery)) OR ((23h* OR 23-h*) ADJ2 (recovery)) OR level-1-area* OR level-one-area* OR transitional-care OR ((high-acuity) ADJ3 (unit* OR room* OR ward* OR beds OR setting* OR area* OR care))).ab,ti,kf. OR ((IC OR ICU OR intensive-care*) ADJ3 ("use" OR utiliz* OR utilis*)).ti.) **AND** (exp Treatment Outcome/ OR exp Mortality/ OR exp Morbidity/ OR exp Postoperative Complications/ OR Intensive Care Units/ OR Patient Outcome Assessment/ OR Outcome Assessment, Health Care/ OR Reoperation/ OR Length of Stay/ OR Patient Discharge/ OR (outcome* OR mortalit* OR morbidit* OR major-adverse-cardiac-event* OR MACE OR complication* OR ((ICU OR IC OR intensive-care* OR critical-care*) ADJ3 (stay OR admi*)) OR reoperat* OR re-operat* OR length-of-stay* OR discharge* OR return-to-theat*).ab,ti,kf.) NOT (news OR congres* OR abstract* OR book* OR chapter* OR dissertation abstract*).pt. NOT (Case Reports/ OR (case-report).ti.) AND english.la. NOT (exp Animals/ NOT Humans/) NOT (*Ambulatory Surgical Procedures/ OR (((ambulator* OR day OR outpatient*) ADJ3 (surg* OR operat*))).ti.)

**Cochrane 4345**

(((((recovery) NEAR/3 (room*)) OR PACU OR ((postoperativ* OR post NEXT operativ* OR postanaesth* OR postanesth* OR post NEXT anaesth* OR post NEXT anesth*) NEAR/6 (care OR recovery) NEAR/6 (unit* OR room* OR ward* OR beds OR setting* OR area* OR admission*)) OR ((extended OR prolong*) NEAR/6 (postoperativ* OR post NEXT operativ* OR postanaesth* OR postanesth* OR post NEXT anaesth* OR post NEXT anesth*) NEAR/6 (recovery OR care OR beds OR setting* OR area* OR admission*)) OR ((high NEXT dependenc* OR high NEXT care*) NEXT/3 (unit* OR room* OR ward* OR beds OR setting* OR area* OR admission*)) OR ((surgical) NEXT/3 (intensive NEXT care* OR IC) NEXT/3 (unit* OR ward* OR room* OR beds OR setting* OR area* OR admission*)) OR ((intermediate) NEAR/6 (care) NEAR/6 (unit* OR room* OR ward* OR beds OR setting* OR area* OR admission*)) OR IMCU* OR ((overnight) NEAR/6 (intensiv*) NEAR/6 (recovery)) OR ((23h* OR 23 NEXT h) NEXT/2 (recovery)) OR level NEXT 1 NEXT area* OR level NEXT one NEXT area* OR transitional NEXT care OR ((high NEXT acuity) NEAR/3 (unit* OR room* OR ward* OR beds OR setting* OR area* OR care))):ab,ti OR ((IC OR ICU OR intensive NEXT care*) NEAR/3 ("use" OR utiliz* OR utilis*)):ti) AND ((outcome* OR mortalit* OR morbidit* OR major NEXT adverse NEXT cardiac NEXT event* OR MACE OR complication* OR ((ICU OR IC OR intensive NEXT care* OR critical NEXT care*) NEAR/3 (stay OR admi*)) OR reoperat* OR re NEXT operat* OR length NEXT of NEXT stay* OR discharge* OR return NEXT to NEXT theat*):ab,ti)) NOT (Trial registry record:pt OR "conference abstract":kw OR ((((ambulator* OR day OR outpatient*) NEAR/3 (surg* OR operat*))):ti)) NOT ("conference abstract":kw OR Trial registry record:pt)

**Web of Science 14241**

TS=(((((recovery) NEAR/2 (room*)) OR PACU OR ((postoperativ* OR post-operativ* OR postanaesth* OR postanesth* OR post-anaesth* OR post-anesth*) NEAR/5 (care OR recovery) NEAR/5 (unit* OR room* OR ward* OR beds OR setting* OR area* OR admission*)) OR ((extended OR prolong*) NEAR/5 (postoperativ* OR post-operativ* OR postanaesth* OR postanesth* OR post-anaesth* OR post-anesth*) NEAR/5 (recovery OR care OR beds OR setting* OR area* OR admission*)) OR ((high-dependenc* OR high-care*) NEAR/2 (unit* OR room* OR ward* OR beds OR setting* OR area* OR admission*)) OR ((surgical) NEAR/2 (intensive-care* OR IC) NEAR/2 (unit* OR ward* OR room* OR beds OR setting* OR area* OR admission*)) OR ((intermediate) NEAR/5 (care) NEAR/5 (unit* OR room* OR ward* OR beds OR setting* OR area* OR admission*)) OR IMCU* OR ((overnight) NEAR/5 (intensiv*) NEAR/5 (recovery)) OR ((23h* OR 23-h) NEAR/2 (recovery)) OR level-1-area* OR level-one-area* OR transitional-care OR ((high-acuity) NEAR/2 (unit* OR room* OR ward* OR beds OR setting* OR area* OR care)))) **AND** ((outcome* OR mortalit* OR morbidit* OR major-adverse-cardiac-event* OR MACE OR complication* OR ((ICU OR IC OR intensive-care* OR critical-care*) NEAR/2 (stay OR admi*)) OR reoperat* OR re-operat* OR length-of-stay* OR discharge* OR return-to-theat*)) NOT ((animal* OR rat OR rats OR mouse OR mice OR murine OR dog OR dogs OR canine OR cat OR cats OR feline OR rabbit OR cow OR cows OR bovine OR rodent* OR sheep OR ovine OR pig OR swine OR porcine OR veterinar* OR chick* OR zebrafish* OR baboon* OR nonhuman* OR primate* OR cattle* OR goose OR geese OR duck OR macaque* OR avian* OR bird* OR fish*) NOT (human* OR patient* OR women OR woman OR men OR man))) AND LA=(English) NOT DT=(Meeting Abstract OR Meeting Summary) NOT TI=(case-report) NOT TI=(((ambulator* OR day OR outpatient*) NEAR/2 (surg* OR operat*)))

**Google Scholar**

"recovery room"|PACU|"postoperative|postanaesthia|postanesthesia care|recovery unit|room|ward"|"intermediate|high|transitional care unit|room|ward" outcome|mortality|morbidity|MACE|complication|reoperation

'recovery room'|PACU|'postoperative|postanaesthia|postanesthesia care|recovery unit|room|ward'|'intermediate|high|transitional care unit|room|ward' outcome|mortality|morbidity|MACE|complication|reoperation
