# Supplementary material for: Outcomes following extended postoperative recovery unit admission in noncardiac surgery: A systematic review and meta-analysis
Source: Eur J Anaesthesiol. 2025 Mar 6;42(5):407–18. doi: 10.1097/EJA.0000000000002145 (PMC11970609; doi:10.1097/EJA.0000000000002145)
Supplement: Supplemental Digital Content [file ejanet-42-407-s002.pdf]

Authors	D1.1	D1.2	D1.3	D1.4	D1.5	D1.6	D1.7	D1.8	D1	D2.1	D2.2	D2.3	D2.4	D2.5	D2	D3.1	D3.2	D3.3	D3	D4.1	D4.2	D4.3	D4.4	D4.5	D4.6	D4.7	D4	D5.1	D5.2	D5.3	D5.4	D5.5	D5	D6.1	D6.2	D6.3	D6.4	D6	D7.1	D7.2	D7.3	D7	Final
Costa-pinto et al.	PY	N		N		N	N		SERIOUS	N			PY		LOW	Y	PY	PN	LOW	Y	N						MODERATE	Y	N	N	NI	NI	LOW	N	N	Y	N	LOW	N	N	Y	MODERATE	MODERATE
Davies et al.	Y	N		N		N	N		SERIOUS	N			PY		LOW	PN	PY	PN	MODERATE	Y	N						MODERATE						NO INFORMATION	N	N	Y	PN	LOW	PN	PN	PN	MODERATE	SERIOUS
Harmse et al.	PY	N		Y	PY	N	PY	Y	MODERATE	N			PY		LOW	Y	PY	PN	LOW	Y	N						MODERATE	PY	N	N	NI	NI	LOW	N	N	Y	PN	LOW	PN	PN	PN	MODERATE	SERIOUS
Heller et al.	PY	N		N		N	N		SERIOUS	PN			PY		LOW	PN	PY	PN	MODERATE	Y	N						MODERATE						NO INFORMATION	N	N	Y	PN	LOW	PN	PN	PN	MODERATE	SERIOUS
Irone et al.	Y	N		PN		PN	N		SERIOUS	PN			Y		MODERATE	PY	PY	PY	MODERATE	PN							MODERATE	NI	NI	NI			NI	PY	PY	PY	PY	MODERATE	PN	PN	PN	MODERATE	SERIOUS
Jerath et al.	PY	N		Y	PY	N	PY	Y	MODERATE	PN			PY		LOW	Y	PY	PN	LOW	Y	N						MODERATE						NO INFORMATION	N	N	Y	PN	LOW	PN	PN	PN	MODERATE	MODERATE
Jerath et al.	PY	N		Y	PY	N	PY	Y	MODERATE	PN			PY		LOW	Y	PY	PN	LOW	Y	N						MODERATE						NO INFORMATION	N	N	Y	PN	LOW	N	N	PY	SERIOUS	MODERATE
Jhanji et al	PY	N		Y	PY	N	PY	Y	MODERATE	PN			PY		LOW	Y	PY	PN	LOW	Y	N						MODERATE						NO INFORMATION	N	N	Y	PN	LOW	PN	PN	PN	MODERATE	SERIOUS
Koning et.	PY	N		Y	PY	N	PY	Y	MODERATE	N			PY		LOW	Y	PY	PN	LOW	Y	N						MODERATE	Y	Y	Y	NI	NI	NO INFORMATION	N	N	Y	PN	LOW	N	Y	PN	SERIOUS	MODERATE
Ludbrook et al.	PY	N		Y	PY	N	PY	Y	MODERATE	PY	PN		Y		MODERATE	Y	Y	N	LOW	Y	N						MODERATE	Y	N	N	NI	NI	LOW	N	N	Y	PN	LOW	N	N	N	LOW	LOW
Ludbrook et al.	Y	PN		N		PY	PY		MODERATE	PN			PY		LOW	Y	Y	N	LOW	Y	N						MODERATE	N	Y	Y	NI	NI	SERIOUS	Y	N	Y	PN	MODERATE	N	N	N	LOW	MODERATE
McIlroy et al.	PY	N		Y	PY	N	PY	Y	MODERATE	N			PY		LOW	Y	Y	N	LOW	Y	N						MODERATE						NO INFORMATION	N	N	Y	PN	LOW	N	N	N	LOW	MODERATE
Paw et al.	Y	N		PY	PY	PY	PY	PY	MODERATE	PN			Y		MODERATE	Y	PY	PY	MODERATE	PN							LOW	NI	NI	NI			NO INFORMATION	PN	PY	Y	PN	LOW	PY	PY	N	MODERATE	MODERATE
Pearse et al.	PY	PN		PY			PY	Y	MODERATE	PN			PY		MODERATE	Y	Y	PY	MODERATE	PN							LOW	Y	Y	NI	NI	NI	LOW	N	PY	Y	PN	LOW	PN	PY	PY	MODERATE	MODERATE
Prin et al.	PY	N		Y	PY	N	PY	Y	MODERATE	N			PY		LOW	Y	Y	N	LOW	Y	N						MODERATE	N	N	N	NI	NI	SERIOUS	N	N	Y	PN	LOW	N	N	N	LOW	MODERATE
Rosenthal et al.	Y	N		PY	PY	PY	PY	PY	MODERATE	PN			PY		MODERATE	PY	PY	PN	MODERATE	PN							LOW	Y	Y	Y	NI	NI	LOW	PN	PY	Y	PY	LOW	PY	PY	PY	MODERATE	MODERATE
Thevathasan et al.	PY	N		Y	PY	N	PY	Y	MODERATE	N			PY		LOW	Y	Y	N	LOW	Y	N						MODERATE						NO INFORMATION	N	N	Y	PN	LOW	N	Y	N	SERIOUS	MODERATE
Turner et al.	PY	PN		PN		N	PN		SERIOUS	N			PY		LOW	N	Y	PN	MODERATE	Y	N						MODERATE						NO INFORMATION	N	N	Y	PN	LOW	N	Y	N	MODERATE	MODERATE
Uzman et al.	PY	N		Y	PY	N	PY	Y	MODERATE	PN			PY		LOW	Y	Y	N	LOW	Y	N						MODERATE						NO INFORMATION	N	N	Y	PN	LOW	N	N	N	LOW	MODERATE
van Tunen et al.	PY	Y	Y	Y	PY	N	PY	Y	SERIOUS	PN			PY		LOW	Y	Y	N	LOW	Y	N						MODERATE						NO INFORMATION	N	N	Y	PN	LOW	N	N	N	LOW	MODERATE
Wickboldt et al.	PY	N		Y	PY	N	PY	Y	MODERATE	PN			PY		LOW	Y	Y	N	LOW	Y	N						MODERATE	Y	N	N	PY	PY	LOW	N	N	Y	PN	LOW	N	N	N	LOW	MODERATE
Wunsch et al.	PY	N		Y	PY	N	PY	Y	MODERATE	PN			PY		LOW	Y	Y	N	LOW	Y	N						MODERATE						NO INFORMATION	N	N	Y	PN	LOW	N	Y	PY	MODERATE	MODERATE

**Authors**	D1.1	D1.2	D1.3	D1.4	D1.5	D1.6	D1.7	D1.8	**D1**	D2.1	D2.2	D2.3	D2.4	D2.5	**D2**	D3.1	D3.2	D3.3	**D3**	D4.1	D4.2	D4.3	D4.4	D4.5	D4.6	D4.7	**D4**	D5.1	D5.2	D5.3	D5.4	D5.5	**D5**	D6.1	D6.2	D6.3	D6.4	**D6**	D7.1	D7.2	D7.3	**D7**	Final
Costa-pinto et al.	PY	N		N		N	N		SERIOUS	N			PY		LOW	Y	Y	PY	LOW	PN							LOW	Y	Y	Y	NI	NI	LOW	PN	PY	Y	PN	LOW	PY	PN	PN	MODERATE	MODERATE
Davies et al.	Y	N		N		N	N		SERIOUS	N			Y		MODERATE	PY	Y	Y	MODERATE	PY	PY						MODERATE	NI	NI	NI			NI	PN	PY	Y	PN	LOW	PN	PN	PN	MODERATE	SERIOUS
Harmse et al.	Y	N		N		Y	N		SERIOUS	N			Y		LOW	PY	Y	PY	MODERATE	Y	Y						SERIOUS	NI	NI	NI			NI	PY	Y	PY	PY	MODERATE	PN	PN	PN	MODERSTE	SERIOUS
Heller et al.	Y	N		N		N	N		SERIOUS	PN			Y		MODERATE	PY	Y	Y	MODERATE	PY	Y						MODERATE	NI	NI	NI			NI	PN	PN	PY	PN	MODERATE	PN	N	PN	MODERATE	SERIOUS
Irone et al.	Y	PN		PN		PN	N		SERIOUS	PN			Y		MODERATE	PY	PY	PY	MODERATE	PN							MODERATE	NI	NI	NI			NI	PY	PY	PY	PY	MODERATE	PN	PN	PN	MODERATE	SERIOUS
Jerath et al. ICM	PY	N		Y	PY	PY	Y	Y	MODERAT	N			PY		LOW	Y	PY	Y	LOW	PY	PY						MODERATE	NI	NI	NI			NI	PN	PY	Y	PY	LOW	PN	PN	PN	MODERATE	MODERATE
Jerath et al. JCA	PY	N		Y	PY	PN	Y	Y	MODERAT	PN			PY		LOW	PY	PY	PY	MODERATE	PY	PY						MODERATE	NI	NI	NI			NI	PN	PY	Y	PY	LOW	Y	PN	PN	MODERATE	MODERATE
Jhanji et al	Y	N		PN		PN	PN		SERIOUS	PN			PY		MODERATE	PY	PY	PY	MODERATE	Y	Y						SERIOUS	NI	NI	NI			NI	PN	PY	PY	PY	MODERATE	PN	PY	PY	MODERATE	SERIOUS
Koning et.	PY	N		PY	PY	PN	PY	Y	MODERAT	N			PY		LOW	Y	Y	PY	LOW	PY	PN						LOW	Y	Y	Y	NI	NI	LOW	PN	PY	Y	PY	LOW	PY	PY	PY	MODERATE	MODERATE
Ludbrook et al. JAMA S	PN								LOW	PY	PN		Y		LOW	Y	Y	PN	LOW	N							LOW	Y	Y	Y	NI	NI	LOW	PN	PY	Y	PY	LOW	PY	PY	PY	LOW	LOW
Ludbrook et al. Anaesthesia	PY	N		Y	Y	PN	PY	Y	MODERAT	PN			PY		MODERATE	Y	Y	PN	LOW	PN							LOW	N	PY	PY	NI	NI	SERIO	PY	PY	PY	PY	MODERATE	PY	PY	Y	MODERATE	MODERATE
McIlroy et al.	Y	PN		Y	Y	PN	Y	PY	MODERAT	N			PY		MODERATE	PY	Y	Y	MODERATE	Y	Y						SERIOUS	NI	NI	NI			NI	PN	PY	Y	PN	LOW	PY	PY	PN	MODERATE	MODERATE
Paw et al.	Y	N		PY	PY	PY	PY	PY	MODERAT	PN			Y		MODERATE	Y	PY	PY	MODERATE	PN							LOW	NI	NI	NI			NI	PN	PY	PY	PN	LOW	PY	PY	N	MODERATE	MODERATE
Pearse et al.	Y	PN		PY			PY	Y	MODERAT	PN			PY		MODERATE	Y	Y	PY	MODERATE	PN							LOW	Y	Y	NI	NI	NI	LOW	N	PY	Y	PN	LOW	PN	PY	PY	MODERATE	MODERATE
Prin et al.	Y	N		PN		PN	PN		MODERAT	PN			PY		MODERATE	Y	PY	PN	MODERATE	PY	PY						MODERATE	Y	PY	PY	NI	NI	LOW	PN	PY	Y	PY	LOW	PN	PY	Y	MODERATE	MODERATE
Rosenthal et al.	Y	N		PY	PY	PY	PY	PY	MODERAT	PN			PY		MODERATE	PY	PY	PN	MODERATE	PN							LOW	Y	Y	Y	NI	NI	LOW	PN	PY	Y	PY	LOW	PY	PY	PY	MODERATE	MODERATE
Thevathasan et al.	Y	N		Y	Y	Y	Y	Y	MODERAT	N			Y		LOW	PN	PN	PN	MODERATE	Y	PY						MODERATE	NI	NI	NI			NI	PN	PY	Y	PY	LOW	N	Y	Y	SERIOUS	MODERATE
Turner et al.	Y	N		N		N	N		MODERAT	PN			PY		MODERATE	PN	PY	PY	MODERATE	Y	PY						MODERATE	NI	NI	NI			NI	PN	Y	PY	PN	LOW	N	N	PY	MODERATE	MODERATE
Uzman et al.	PY	PN		N		PN	N		MODERAT	N			PY		MODERATE	PY	PN	PN	MODERATE	Y	PN						MODERATE	NI	NI	NI			NI	PY	PY	Y	PY	MODERATE	N	N	Y	MODERATE	MODERATE
van Tunen et al.	PY	Y	PY	Y	PY	PY	N		SERIOUS	PN			PY		LOW	PY	Y	N	MODERATE	PN							MODERATE	NI	NI	NI			NI	PY	PY	Y	PY	LOW	PY	PN	PY	MODERATE	MODERATE
Wickboldt et al.	PY	N		Y	Y	N	Y	Y	LOW	N			Y		LOW	PY	PY	PN	MODERATE	PN							LOW	Y	Y	Y	PY	PY	LOW	PN	PY	Y	PY	LOW	N	Y	Y	MODERATE	MODERATE
Wunsch et al.	Y	N		Y	Y	PN	Y	Y	MODERAT	Y	PY	PY	PY	Y	MODERATE	PY	PN	PN	MODERATE	Y	PN						MODERATE	NI	NI	NI			NI	PY	PN	Y	PY	LOW	PY	Y	Y	MODERATE	MODERATE
