# Supplementary figures and images for: Outcomes following extended postoperative recovery unit admission in noncardiac surgery: A systematic review and meta-analysis
Source: Eur J Anaesthesiol. 2025 Mar 6;42(5):407–18. doi: 10.1097/EJA.0000000000002145 (PMC11970609; doi:10.1097/EJA.0000000000002145)

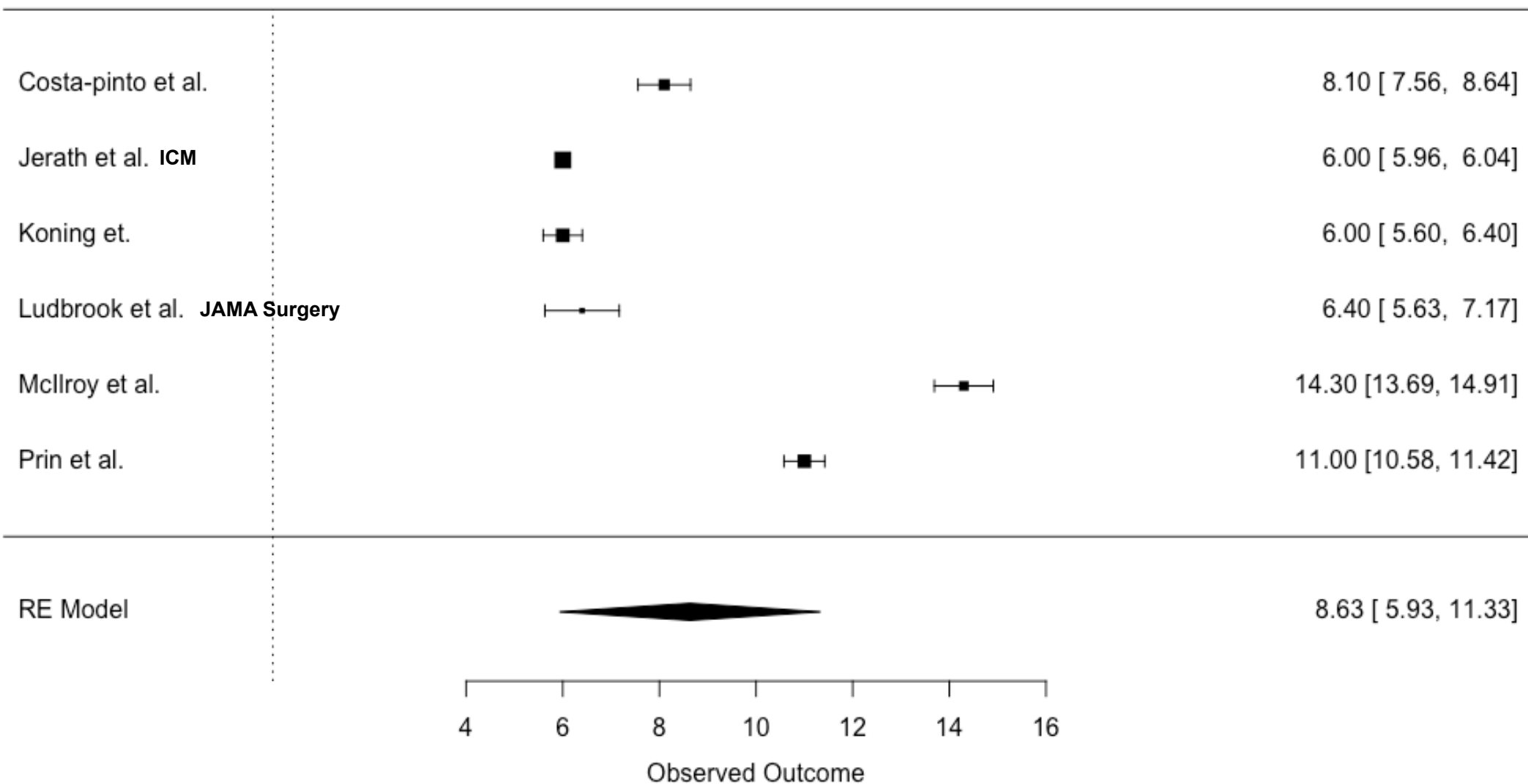

Appendix 2 Pooled Hospital Length of Stay in days, median [95% CI]

Supplement: Supplemental Digital Content [file ejanet-42-407-s003.pdf]
